# Graft-Versus-Host Disease After Liver Transplantation: Diagnostic and Therapeutic Challenges

**DOI:** 10.7759/cureus.110988

**Published:** 2026-06-16

**Authors:** Jerry Qi, Kareem Latif, Leo Sakai, Nidhi Kejriwal, Amy Schill

**Affiliations:** 1 Internal Medicine, Loma Linda University Medical Center, Loma Linda, USA

**Keywords:** case report, graft-versus-host disease, gvhd, immunosuppression, jak inhibitor, liver transplantation, ruxolitinib, transplant complication

## Abstract

Graft-versus-host disease (GVHD) is a rare but severe complication following solid organ transplantation, with limited literature guiding diagnosis and management. We present a 55-year-old man with alcohol-associated end-stage liver disease who developed GVHD approximately one month after orthotopic liver transplantation (LT). He was initially admitted for persistent fever and concern for sepsis, with subsequent development of progressive pancytopenia and a non-pruritic maculopapular rash with mucosal involvement. Skin biopsy performed on hospital day 10 supported the diagnosis of GVHD, and donor chimerism testing demonstrated 70% donor-derived CD3+ lymphocytes. He improved with high-dose intravenous methylprednisolone and remained clinically stable on ruxolitinib maintenance therapy at two-month post-discharge follow-up. GVHD following LT should be considered early in patients with non-specific presentations, such as unexplained fever, cytopenias, rash, or mucosal findings. Early recognition, tissue biopsy, and chimerism testing may facilitate timely intervention and improve outcomes in this rare condition associated with high mortality.

## Introduction

Graft-versus-host disease (GVHD) is a well-recognized complication of hematopoietic stem cell transplantation (HSCT) but is rarely observed following solid organ transplantation (SOT), including liver transplantation (LT) [1-7]. The reported incidence of GVHD after LT ranges from approximately 1% to 2% across published series, with a national analysis of over 88,000 LTs estimating an incidence of 1% [6]. The condition carries a high mortality rate, with six-month mortality reported at 73% in a comprehensive literature review [1]. GVHD typically develops within 2-12 weeks after SOT, with a median onset of approximately 28-30 days after transplantation, though delayed presentations beyond this window have also been reported [1,2]. Due to its rarity, there is limited consensus regarding diagnostic criteria and optimal treatment strategies for post-LT GVHD.

The pathogenesis of GVHD in LT recipients is thought to involve immunocompetent donor-derived T lymphocytes within the transplanted liver that become activated against immunosuppressed host tissues, causing immune-mediated organ dysfunction [1]. The liver is a lymphocyte-rich organ, and donor T cells that engraft in the recipient can mount an alloreactive response targeting the skin, gastrointestinal tract, and bone marrow [8]. Immunosuppressive regimens used to prevent graft rejection may also paradoxically facilitate the development of GVHD by impairing the recipient’s ability to reject donor lymphocytes [2]. Recognized risk factors include recipient-donor age discrepancy, HLA class I similarity between donor and recipient, and certain immunosuppressive regimens [9].

Clinically, GVHD following LT typically presents with non-specific, multisystem manifestations involving the skin, gastrointestinal tract, and bone marrow. Common clinical features include fever, rash, diarrhea, and pancytopenia. These symptoms overlap substantially with infectious, medication-related, and rejection-related processes in post-transplant patients, often leading to delayed diagnosis and contributing to poor outcomes.

There are no standardized diagnostic criteria specific to post-LT GVHD, with the diagnostic approach largely extrapolated from HSCT-associated GVHD frameworks. Diagnosis relies on a combination of clinical features, histopathological findings, and chimerism analysis [8,10]. Evidence of donor chimerism in peripheral blood or tissue, detected by short tandem repeat (STR) PCR, supports the diagnosis by confirming the presence of donor-derived lymphocytes in the recipient [8,11]. Early recognition, therefore, depends on a high index of clinical suspicion, with prompt biopsy and chimerism testing in post-LT patients who develop unexplained fever, cytopenias, rash, or mucosal findings. We report a case of GVHD following LT presenting with non-specific symptoms, highlighting the diagnostic challenges and management of this rare and frequently fatal condition.

## Case presentation

A 55-year-old man with a history of alcohol-associated end-stage liver disease underwent orthotopic LT with an uneventful postoperative course. Induction immunosuppression consisted of basiliximab, and maintenance immunosuppression included tacrolimus, mycophenolate mofetil (MMF), and prednisone. Prophylactic medications included trimethoprim-sulfamethoxazole for *Pneumocystis jirovecii* prophylaxis and valganciclovir for cytomegalovirus prophylaxis.

On day 31 after transplant, the patient presented to the emergency department with persistent fever and right-sided tooth pain. Two days prior to his presentation, his prednisone dose had been decreased as part of a planned steroid taper, and MMF was discontinued due to gastrointestinal intolerance. Initial laboratory studies demonstrated mild anemia with otherwise unremarkable cell counts (Table 1). Physical examination was notable for poor dentition and a soft, non-tender, non-distended abdomen. He was admitted for further evaluation of persistent fever and concern for sepsis.

**Table 1:** Hematologic trends during hospitalization. Reference ranges reflect institutional standards.

Laboratory test	Reference range	On admission	Hospital day 6
White blood cell count (WBC), ×10^9^/L	4.80-11.80	6.3	1.47
Hemoglobin (Hgb), g/dL	11.0-16.0	10.1	6.8
Platelet count, ×10^9^/L	140-340	134	41
Absolute neutrophil count (ANC), ×10^9^/L	1.58-8.3	N/A	1.4
Eosinophil count, ×10^9^/L	0.00-0.80	N/A	0.32

The patient underwent dental extraction for a periapical abscess and was treated empirically with intravenous broad-spectrum antibiotics. Despite these interventions, he continued to have high-grade fevers. No diarrhea or other gastrointestinal symptoms were present. Additional infectious workup, including blood cultures, urine cultures, chest radiography, and abdominal CT scans, was otherwise negative.

On hospital day 6, the patient developed progressive pancytopenia with leukopenia, anemia, and thrombocytopenia (Table 1). Concurrently, he developed a non-pruritic, erythematous maculopapular rash involving the trunk and proximal extremities without involvement of the palms or soles and without desquamation. On hospital day 8, he developed severe oral mucositis with ulcerations involving the lips, oral mucosa, and hard palate.

The initial differential diagnosis included drug reaction with eosinophilia and systemic symptoms (DRESS), Stevens-Johnson syndrome, and GVHD. Peripheral eosinophil count remained within normal limits, which made DRESS less likely. Given the combination of progressive multi-lineage cytopenias, mucocutaneous involvement, and negative infectious workup, dermatology was consulted, and a skin biopsy was performed on hospital day 10. Given the high clinical suspicion for GVHD, high-dose pulse intravenous methylprednisolone 500 mg daily was initiated empirically for three days prior to confirmatory results, with resolution of fevers and improvement of the cutaneous rash and oral ulcers.

Histopathology of the skin biopsy demonstrated features of vacuolar interface dermatitis, including scattered dyskeratotic keratinocytes with adjacent lymphocytic satellitosis, consistent with GVHD. Chimerism analysis confirmed the diagnosis, demonstrating 1.7% total donor cells, 70% donor-derived CD3+ lymphocytes, and 0.6% donor-derived myeloid cells. The high proportion of donor-derived CD3+ T lymphocytes relative to total donor chimerism was consistent with selective donor T-cell expansion, which is central to the pathogenesis of GVHD. Following clinical improvement with high-dose methylprednisolone, the patient was transitioned to an oral prednisone taper over four weeks and initiated on ruxolitinib 5 mg twice daily. At the two-month post-discharge follow-up, he remained clinically stable on ruxolitinib 5 mg twice-daily maintenance therapy, with complete resolution of rash, oral ulcers, and cytopenias. Repeat chimerism testing demonstrated a decline in donor-derived CD3+ cells from 70% to 0.8%, consistent with near-resolution of GVHD.

## Discussion

This case illustrates the diagnostic difficulty of GVHD following LT, particularly in the setting of non-specific and evolving clinical features. In this patient, persistent fever with initially unrevealing infectious studies preceded the development of progressive pancytopenia, cutaneous eruption, and mucosal involvement. The delayed appearance of these hallmark findings highlights how GVHD may initially present without a unifying diagnosis, increasing the risk of diagnostic delay [1-3]. The timeline in this case, with symptom onset approximately four weeks after LT, is consistent with previously published reviews reporting median times to GVHD onset of approximately 28-30 days [1,2].

A major challenge in post-LT GVHD is distinguishing it from more common post-transplant complications. Fever and cytopenias are frequently attributed to infection, medication toxicity, or sepsis, while rashes may be misinterpreted as drug reactions [1,2,8,9]. In this case, the broad differential diagnosis appropriately included DRESS and Stevens-Johnson syndrome. However, the absence of peripheral eosinophilia made DRESS less likely, and the combination of progressive multi-lineage cytopenias with mucocutaneous involvement in the setting of a negative infectious workup prompted tissue biopsy and chimerism testing, which were critical in establishing the diagnosis. The clinical course in this patient reinforces the importance of maintaining diagnostic vigilance when post-transplant patients develop unexplained, multi-organ findings despite appropriate initial management. Early biopsy and chimerism testing should be considered in post-LT patients presenting with unexplained cytopenias, fever, rash, or mucosal findings.

Notably, the discontinuation of MMF and reduction of prednisone shortly before symptom onset may have altered the immunologic balance and contributed to the development of GVHD in this patient, though a causal relationship cannot be established from a single case. In a published registry analysis of 77,416 LT recipients, MMF maintenance was associated with a decreased risk of fatal GVHD [6]. The concept that reductions in immunosuppression may unmask or facilitate donor T-cell activation is supported by the observation that the recipient’s impaired ability to reject donor lymphocytes is central to GVHD pathogenesis. This underscores the importance of careful immunosuppression management in the early post-transplant period, while recognizing that MMF was discontinued in this case due to gastrointestinal intolerance.

Management of GVHD following LT remains challenging and is not guided by standardized treatment algorithms specific to the post-SOT setting. Therapeutic approaches are largely extrapolated from HSCT-associated GVHD, although important differences exist. Notably, the liver graft itself is typically spared in post-LT GVHD, and immunosuppression management must balance GVHD treatment against the risk of graft rejection [1,2,8,12,13]. A key paradox in post-SOT GVHD management is whether to escalate or reduce immunosuppression. Increasing immunosuppression aims to suppress donor T-cell activity, while reducing immunosuppression may allow the recipient’s immune system to reject donor lymphocytes. Current evidence has not demonstrated a clear survival benefit with either approach, and treatment decisions must be individualized based on the extent of organ involvement and clinical trajectory [1,2,14,15]. Corticosteroids are typically initiated as first-line therapy, though responses are often incomplete or transient [1,3]. Additional agents described in the literature include antithymocyte globulin, interleukin-2 receptor antagonists, tumor necrosis factor-α antagonists, calcineurin inhibitor adjustment, and, more recently, JAK inhibition. As with this patient, treatment is often started on clinical suspicion prior to confirmatory testing given the potential consequences of diagnostic delay.

In this case, high-dose corticosteroids resulted in rapid clinical improvement, and adjunctive therapy with ruxolitinib was associated with sustained resolution of symptoms at two-month follow-up. Ruxolitinib, a selective JAK1/JAK2 inhibitor, is FDA-approved for steroid-refractory acute GVHD after HSCT, with demonstrated efficacy in trials [12,16]. However, evidence supporting JAK inhibition specifically in post-SOT GVHD remains limited to case reports and small series. Endo et al. first described durable complete remission of post-LT GVHD with ruxolitinib, and Jacobs et al. reported clinical improvement in three SOT recipients treated with ruxolitinib, with additional favorable outcomes described in liver and lung transplant recipients [7,17,18]. Despite these favorable reports, overall mortality from post-LT GVHD remains high [1,2]. The favorable response observed in this case, including resolution of rash, oral ulcers, and cytopenias, along with a decline in donor-derived CD3+ cells from 70% to 0.8%, adds to this small but growing body of evidence supporting a potential role for ruxolitinib in selected patients with post-SOT GVHD. However, its efficacy in this context should be interpreted cautiously given the case-based nature of the evidence.

## Conclusions

Overall, this case emphasizes the need for early consideration of GVHD in liver transplant recipients presenting with unexplained fever, cytopenias, and mucocutaneous involvement. Early recognition and a low threshold for biopsy, confirmatory testing, and treatment may facilitate prompt interventions and improve outcomes in this rare but potentially fatal complication of organ transplantation. In this patient, timely biopsy, donor chimerism testing, and empiric corticosteroid initiation were key steps that supported the diagnosis and treatment. Although clinical improvement was sustained with corticosteroids followed by ruxolitinib, evidence supporting ruxolitinib in post-SOT GVHD remains limited to case reports and small series; findings from this single case with limited follow-up should be interpreted with caution. Further studies are needed to refine diagnostic approaches and define management strategies for post-LT GVHD.
